# Genetic diversity and colony structure of *Tapinoma melanocephalum* on the islands and mainland of South China

**DOI:** 10.1002/ece3.4065

**Published:** 2018-05-02

**Authors:** Chunyan Zheng, Fan Yang, Ling Zeng, Edward L. Vargo, Yijuan Xu

**Affiliations:** ^1^ Department of Entomology South China Agricultural University Guangzhou China; ^2^ Department of Entomology Texas A&M University College Station Texas

**Keywords:** colony structure, genetic diversity, island, mainland, *Tapinoma melanocephalum*

## Abstract

**Aim:**

*Tapinoma melanocephalum* is listed as one of the most important invasive pest species in China. Information regarding the patterns of invasion and effects of geographic isolation on the population genetics of this species is largely lacking.

**Location:**

South China.

**Methods:**

To address this problem, we genotyped 39 colonies (two colonies were collapsed due to genetic similarity) using microsatellite markers and mitochondrial DNA sequencing to compare colony genetic structure of *T. melanocephalum* on the mainland and islands of South China.

**Results:**

An analysis of the colony genotypes showed that the genetic diversity of the mainland population was slightly higher than that of the island populations but not significantly so. However, the observed heterozygosity on Shangchuan Island (SCD) was significantly lower than that of the other colonies. We also found six haplotypes in 111 mitochondrial DNA COI sequences. The relatedness (*r*) value between colonies of SCD was 0.410, higher than that of the other populations. The genetic clusters among colonies were not related to geographic locations and exhibited admixture likely due to frequent human‐mediated dispersal associated with trade between the mainland population and the islands. Pairwise *F*_ST_s between populations showed differentiation among mainland populations, while SCD displayed high levels of divergence (*F*_ST_ > 0.15) from most mainland populations. There was no significant isolation by distance among colonies. Most populations showed signs of a bottleneck effect.

**Main conclusions:**

Our study suggests that there was no significant difference in the genetic diversity among the islands and the mainland; however, the lower genetic diversity, the higher degree of genetic divergence from other colonies, and the higher relatedness among nestmates made the SCD population stand out from all the others.

## 1 INTRODUCTION

Biological invasions are considered a threat to global biodiversity, and invasive ants are among the most successful invaders (Ross & Keller, 1995). The successful invasion of species is closely related to ecological, evolutionary, and reproductive factors as well as migration ability (Parker, Rodriguez, & Loik, 2003). In new environments, recently established populations of exotic species experience decreased genetic variability due to bottlenecks, which leads to inbreeding depression (Allee, Park, Emerson, Park, & Schmidt, 1949; Linhart & Grant, 1996).

*Tapinoma melanocephalum* (Fabricius) (Hymenoptera: Formicidae), the ghost ant, is a ubiquitous invasive species and widely distributed in the tropical and subtropical latitudes worldwide, and even invades temperate zones through commercial activity and human trade (Espadaler & Espejo, 2002; Wilson, 1967). *T. melanocephalum* is not only a household‐infesting ant (Nickerson, Bloomcamp, & Fasulo, 2004), but also an agriculture pest associated with hemipteran species, such as mealybugs and scales (Fowler, Bernardi, Delabie, Forti, & Pereira‐da‐Silva, 1990; Venkataramaiah & Rehman, 1989). It was first known to invade China in ca. 1929. Currently, it is frequently found in disturbed habitats where it nests in old branches, dry grass clumps, moist grass, and plant stems. It is also likely to be found in rotten wood, soil, and decayed parts of trees or beneath bark as well as in the walls of houses and in plant pots. It has successfully spread through several provinces in South China and some areas of North China through human assistance (Wetterer, 2009).

In their introduced ranges, changes in colony structure have facilitated success of many invasive social insects, such as the ant *Anoplolepis gracilipes* and *Linepithema humile* (Corin, Abbott, Ritchie, & Lester, 2007; Thomas, Becker, Abbott, & Feldhaar, 2010). Invasive social insects often form supercolonies containing in some cases billions of workers and thousands of queens spread over hundreds of square kilometers (Holway, Lach, Suarez, Tsutsui, & Case, 2002; Holway & Suarez, 2004). Like other invasive ants, colonies of *T. melanocephalum* are polygynous, usually unicolonial; mating is intranidal; colonies are mostly founded by budding; and swarming is generally lacking (Smith & Whitman, 1992). Workers within unicolonial societies, or supercolonies, can move freely among different nests without aggression. Different supercolonies can exist near each other and exhibit distinct colony boundaries (Waldman, Frumhoff, & Sherman, 1988). On Christmas Island, the invasive ant *Anoplolepis gracilipes* established two supercolonies with an unusual reproductive strategy resulting in workers that were heterozygous at all microsatellite loci examined (Thomas et al., 2010).

There have been several hypotheses to explain unicoloniality of invasive ants from a genetic perspective. Tsutsui, Suarez, Holway, and Case (2000) reported that unicoloniality of invasive ants in their introduced regions may result from loss of genetic diversity. Invasive populations showed lower genetic diversity due to a genetic bottleneck. Lower genetic variability may result in a change in the nestmate recognition system, leading to decreased aggression (Bourke & Franks, 1995; Tsutsui et al., 2000; Vásquez & Silverman, 2008). Another mechanism for unicolonial formation has been proposed for *Linepithema humile* by “genetic cleansing.” Giraud, Pedersen, and Keller (2002) hypothesized that colonies could share common recognition alleles. These recognition signals might gradually mix with colonies or supercolonies due to reduced interpopulation aggression and increased encounter rates between colonies.

Genetic diversity has played an important role in the evolutionary changes of species in response to changing environmental stress (Frankel & Soulé, 1981), and it is one of the three levels of biodiversity promoting the long‐term survival of a species (Frankham, Briscoe, & Ballou, 2002; Hedrick & Kalinowski, 2000; Morley et al., 2004). The ability of invasive ants to resist environmental stress and adapt to changes in the environment is dependent on genetic diversity (Beardmore, Mair, & Lewis, 1997). Therefore, studies on genetic diversity would be helpful for understanding the evolution and adaptation of invasive ants in their introduced environments (Sakai et al., 2001). Genetic constraints potentially influence initial colonization and will also influence the rate of spread (Antonovics, 1976; Sakai et al., 2001). Islands are isolated from nearby mainland by large water barriers and are usually smaller in area than the mainland (Losos & Ricklefs, 2009a). Geographic isolation of invasive species from the mainland results in experiencing different environmental conditions, food resources, and biological competitors and predators. In addition, the water surrounding islands acts as a geographic barrier to dispersal that limits gene flow both between island populations and between island and mainland populations (Barnett et al., 2013; Duffie, Glenn, Vargas, & Parker, 2009). Previous bottlenecks or continued isolation from the mainland can also decrease genetic diversity of invasive species in islands (Losos & Ricklefs, 2009b; Pimm, Diamond, Reed, Russell, & Verner, 1993).

Here, we explore the genetic and environmental (geographic isolation) factors in introduced areas to determine and compare the population genetic structure and colony characteristics of *T. melanocephalum* between island and mainland regions using six microsatellite loci and COI sequence data, and examine the relationship between two levels of population structure. The aim of this study is to characterize the breeding structure of the colonies and to investigate the relationships among populations and the mechanism of population spread.

## 2 MATERIALS AND METHODS

### 2.1 Sampling and study sites

**Figure 1:**
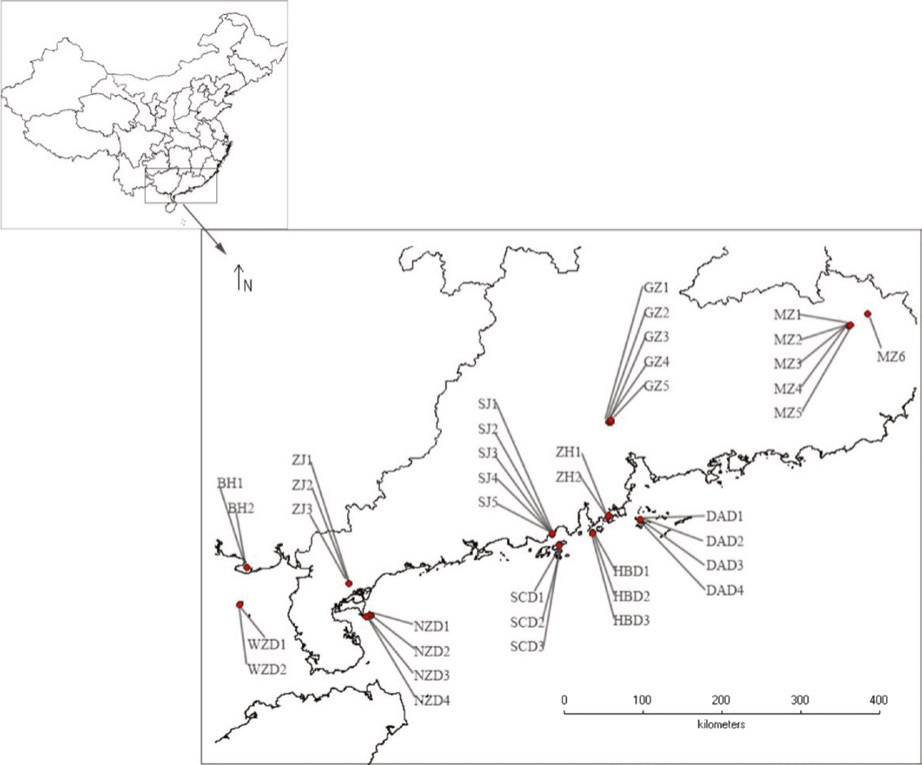
Locations of the five island populations and six mainland populations of *Tapinoma melanocephalum* examined in this study. The map was generated by DIVA‐GIS version 7.1

All samples of *T. melanocephalum* were collected from April to June 2015 using 50‐ml plastic vials baited with chicken sausage (Shuanghui Group CO., LTD, Luohe, China). *T. melanocephalum* samples were collected at 23 and 16 points from the mainland and islands, respectively (Figure 1). Every two collection points were separated by at least 40 m. The sample collection points from six mainland populations were in Beihai (BH, two points: BH1 and BH2), Zhanjiang (ZJ, three points: ZJ1, ZJ2, and ZJ3), Shanju (SJ, five points: SJ1–SJ5), Guangzhou (GZ, five points: GZ1–GZ5), Zhuhai (ZH, two points: ZH1 and ZH2), and Meizhou (MZ, six points: MZ1–MZ6). On the islands, samples were collected from Weizhou (WZD, two points: WZD1 and WZD2), Naozhou (NZD, four points: NZD1–NZD4), Shangchuan (SCD, three points: SCD1–SCD3), Hebao (HBD, three points: HBD1–HBD3), and Dong'ao (DAD, four points: DAD1–DAD4) populations. The GPS coordinates were obtained by DIVA‐GIS 7.1 software (see Table S1). After collection, the samples were preserved in 90% ethanol and stored at 4°C until DNA extraction.

### 2.2 Molecular techniques

DNA from at least 18 workers from each collection point was extracted using the TIANamp Micro DNA Kit (TianGen Biology CO, Ltd., Beijing, China). In total, 778 workers were genotyped at six microsatellite loci: locus 911, locus 1315, locus 364, locus 434, locus 592, and locus 1178 (GenBank accession nos.: KX641151–KX641156). The polymerase chain reaction (PCR) amplification was performed in 25 μl containing 1.5 μl of DNA extract (25 ng/μl), 1 μl of each primer (10 μmol/L; synthesized by Sangon Biotech (Shanghai) Co., Ltd., Shanghai, China), 12.5 μl Premix rTaq (TaKaRa, Japan), and 9 μl of ddH_2_O. The cycling conditions for the amplification were as follows: 3 min at 94°C followed by 35 cycles of 30 s at 94°C, 30 s at annealing temperature (see Table 1 for annealing temperature for each primer), 45 s at 72°C, and an extension of 5 min at 72°C. The PCR products were diluted 1:10 with 1× dilution buffer (5 × 930 DNA Dilution Buffer) and separated by a Fragment Analyzer^™^ automated capillary electrophoresis system (Advanced Analytical Technologies, Inc., USA). The alleles were scored using the software PROSize 2.0 (Advanced Analytical Technologies, Inc., USA).

**Table 1:** Description of the six primers used for genotyping 778 individuals

Locus	Repeat	Primer sequence forward Primer sequence reverse	*T*_m_ (°C)	Product size	Range (bp)	GenBank accession no.	Mainland		Island		PHWE
							A	AR	A	AR	
Locus 911	(CA)14	GCCTCGTCAAGAGTGGTCTC	54	259	236–269	KX641151	31	30.46	25	25	*p* < .001
		GGAAAGCAGCAATTTTCTCG									
Locus 1178	(TG)15	CACAGTACCCTGGAGGCATT	53	187	490–519	KX641152	25	24.641	28	28	*p* = .172
		CGTGAGAGAAATTTGCGTGA									
Locus 1315	(TATAC)22	GCATGTGTGCAGTCTCGAAT	54	261	261–348	KX641153	14	13.956	15	14.994	*p* = .003
		GGGTCTGATGGAATACCGTG									
Locus 434	(AC)28	AGCTCGGCTGATTCGTTATG	52	129	106–139	KX641154	22	21.885	19	19	*p* < .05
		TTCTTTTCACTCGTGTTGCG									
Locus 364	(CA)27	GCACTTTTCAGTGCCCATTT	53	330	91–144	KX641155					*p* < .001
		CTGCTATTACTGGCACGCTG									
Locus 592	(AC)18	CGAACGAATCCTTGGTCAAT	53	221	395–430	KX116456	19	18.852	18	17.994	*p* = .748
		TACATCGTCCGACACAGCTC									
Mean							22.2A	21.9588b	21A	20.9976b	*p* < .001

The variability at five microsatellite loci in *Tapinoma melanocephalum* was tested from mainland populations and island populations. The number of alleles (A) and allele richness (AR) were estimated among all samples. PHWE shows the *p* value for the test of Hardy–Weinberg equilibrium. Data with lowercase or uppercase letter indicate no significant difference between island populations and mainland populations using an independent *t* test (*p* > .05).

The partial cytochrome oxidase subunit I (COI) gene of the mitochondrial DNA was amplified and sequenced from 111 individuals from all colonies using the primers LCO1490 (5′‐GGT CAACAAATCATAAAGATATTGG‐3′) and HCO2198 (5′‐TAAACTTCAGGGTGACCAAAAAATCA‐3′) (modified from Hebert, Ratnasingham, & de Waard, 2003; GenBank accession number: JQ913600). The PCR amplification reaction and cycling followed the method described by Chiotis, Jermiin, and Crozier (2000), and the PCR products were sent for sequencing to Sangon Biotech Co., Ltd (Shanghai, China). All sequences were edited in DNAStar. If necessary, errors were manually corrected and then aligned using MEGA 6.0 software after blasting in NCBI. Based on a 610‐bp fragment, the genetic diversity of the COI gene was estimated.

### 2.3 Colony boundary test

To determine whether workers collected from different points could be attributed to the same colony, genotypic differentiation with pairs of samples collected from the different geographic points from each site was performed using the log‐likelihood G test in GENEPOP version 4.5.1 (Raymond & Rousset, 1995). The *p* values were detected using Fisher's probability with the standard Bonferroni correction. Two sampling locations were considered to be different colonies if the genotypic differentiation of workers from them was shown to be significantly different (Deheer & Vargo, 2004; Dronnet, Chapuisat, Vargo, & Bagnères, 2005).

### 2.4 Genetic diversity testing

The Hardy–Weinberg equilibrium for each locus and linkage disequilibrium (LD) for each pair of loci were tested by GENEPOP version 4.5.1 (Raymond & Rousset, 1995). To avoid high relatedness within colonies, we randomly selected one individual from each colony to test. For linkage disequilibrium testing, we performed 10 repetitive calculations by taking 10 different individuals from each colony. The *p* values were tested using the Bonferroni correction for multiple tests (Rice, 1989). FreeNA software was used for checking the null allele for each locus and colony with the exception maximization (EM) algorithm (Chapuis & Estoup, 2007; Dempster, Laird, & Rubin, 1977). The number of alleles and the allele richness in each colony and population were analyzed by FSTAT 2.9.3 (Goudet, 1995). The observed heterozygosity and the Shannon index of each colony or population were estimated by GENALEX 6.5 software (Peakall & Smouse, 2006). The Shannon index is a measure of the genetic diversity of a population that is estimated based on the allele number and frequency, and the resulting values ranged from 0 (low diversity) to 4.6 (high diversity; Colwell, Mao, & Chang, 2004). When we compared the genetic diversity among all island and mainland populations, our results were estimated at the population levels of the island and mainland, instead of at the colony level. Significant differences were determined by SPSS 14.0 using an independent *t* test. The observed heterozygosity of all populations did not have similar variances and was compared using a Kruskal–Wallis test, and if the differences were significant, multiple comparisons of means were performed with the Mann–Whitney test. In addition, the haplotype diversity of each colony was detected by DnaSP (Rozas, Sánchez‐DelBarrio, Messeguer, & Rozas, 2003).

### 2.5 Genetic structure of colonies and populations

To determine the genetic relationships among populations, each colony was considered a subpopulation in each geographic site (similar to that reported by Vargo, 2003) and was subjected to an independent simulation run by STRUCTURE software based on the admixture model and an allele frequency‐correlated model, with different genotypes of individuals allocated to different genetic clusters (Evanno, Regnaut, & Goudet, 2005; Pritchard, Wen, & Falush, 2010). Here, we ran all individuals genotyped at the population level, taking the first individual genotyped per colony. We performed 10 runs for each value of K with the simulated number of geographic populations from 1 to 11 with 5,000 runs of MCMC after a burn‐in period of 10,000 runs that was discarded after burning. After running, we uploaded the results file into STRUCTURE HARVESTER (http://taylor0.biology.ucla.edu/structureHarvester/#) to determine the most likely number of genetic clusters (K) using the method of Evanno et al. (2005).

The coefficient of relatedness among workers was estimated within all 37 colonies sampled and worker nestmates of colonies from 11 geographic populations for genetic analysis using FSTAT 2.9.3, following Queller and Goodnight (1989) using all individual genotypes as the reference population. The index of relatedness *R* weights each allele inversely by its frequency in the population so that rare alleles are given a relatively higher weight.

### 2.6 Isolation by distance

To estimate the degree of genetic differentiation at the population level at this scale, we calculated the pairwise F_*ST*_ *s* for 11 geographic populations with one individual from each colony using GENEPOP version 4.5.1. The differentiation index was interpreted in a standard manner as follows: a low degree of genetic differentiation (0 ≤ *F*_ST_ < 0.05); a medium degree of genetic differentiation (0.05 ≤ *F*_ST_ < 0.15); a high degree of genetic differentiation (0.15 ≤ *F*_ST_ ≤ 0.25); and a very high degree of genetic differentiation (*F*_ST_ > 0.25; Rousset, 1997). To assess the colony level of genetic differentiation related to the geographic distance, we considered colonies in the island and mainland areas separately as population genetic units instead of geographic populations based on site. The correlation coefficients between *F*_ST_/(1 − *F*_ST_) and the natural log of the geographic distance (km) were run using GENEPOP version 4.5.1 with Mantel tests (Raymond & Rousset, 1995).

### 2.7 *F*‐statistics analysis

To assess genetic variation among the island and mainland populations, we performed a three‐level analysis of *F*‐statistics (Weir & Cockerham, 1984). In this part, only five loci (locus 434, locus 592, locus 911, locus 1178, and locus 1315) were used in the *F*‐statistics analysis. We followed the approach of Thorne et al. (1999) partitioning genetic variation into three levels: individual, colony, and region. Here, regions included five island populations (14 colonies) and six mainland populations (23 colonies). Arlequin v3.5.1.3 was used to perform an analysis of molecular variance (AMOVA) with three different levels, and different fixation indices were obtained: *F*_INDIVIDUAL‐COLONY_, *F*_COLONY‐REGION_, and *F*_REGION‐TOTAL_. The *p* value was calculated by 10,000 replications (Schneider, Roessli, & Excoffier, 2000).

### 2.8 Genetic bottleneck test

The program BOTTLENECK 1.2.02 was used to detect whether the populations recently experienced bottlenecks (Piry, Luikart, & Cornuet, 1999). The infinite allele model (IAM) combined with the stepwise mutation model (SMM) based on two statistical tests, the sign test and the Wilcoxon signed‐rank test, were performed to test whether there was significant heterozygosity excess at all loci. The tests were performed on replicate data sets for each population. In each population, we used at least 18 individuals for each colony. Totally, 3,112 tests (778 individuals) were performed for all populations (778 resampled data sets × two tests × two models for mutation–drift equilibrium). The significance of the bottleneck tests on all data sets was examined using Fisher's combined probability test.

## 3 RESULTS

### 3.1 Colony boundaries and breeding structure

The results of the tests of genetic differentiation between collection points indicate the presence of 14 distinct colonies among the island samples. A total of four collection points from the NZD population were grouped into three colonies (significance of G test between NZD2 and NZD3 points which were 500 m apart, *p *=* *.1248; remaining pairs of colonies, all *p *<* *.001). For the SCD population, the SCD2 and SCD3 points, which were 80 m apart, were grouped into one colony (significance of *G* test, *p *=* *.1186). All collection points from the HBD (three colonies), DAD (four colonies), and WZD (two colonies) populations were shown to be unique colonies (*G* test, *p *<* *.001 for all). For the mainland populations, each pair of collection points was significantly differentiated (*G* test, *p *<* *.001 for all) and therefore considered separate colonies (Table S1). An analysis of the number of alleles per colony indicated that all of 37 colonies of mainland and island populations had more than four alleles and was consistent with their polygyne social structure that has been previously reported (Wetterer, 2009; Table 2).

**Table 2:** Colony genetic diversity for 11 geographic populations using five microsatellite loci and mitochondrial DNA haplotypes per colony

Colony	Microsatellite analysis					COI haplotype
	A	AR	HO	I	*r*	
Shanju	6.2	6.0444	0.4691 ± 0.1619abc	1.3306	0.303 ± 0.033	
COLSJ1	6.4000	6.2738	0.4400	1.3661		A
COLSJ2	5.4000	5.2966	0.5200	1.3596		A
COLSJ3	5.0000	4.9106	0.4700	1.2479		A
COLSJ4	7.0000	6.7860	0.4300	1.3186		E
COLSJ5	7.2000	6.9552	0.4856	1.3609		A
Zhuhai	5.4	5.3036	0.5650 ± 0.0350a	1.3420	0.293 ± 0.072	
COLZH1	5.8000	5.7320	0.6000	1.4615		B
COLZH2	5.0000	4.8752	0.5300	1.2226		A
Meizhou	5.93	5.7829	0.5467 ± 0.0212ab	1.3390	0.274 ± 0.027	
COLMZ1	5.6000	5.4918	0.5000	1.2224		A
COLMZ2	6.0000	5.8338	0.6100	1.3744		C
COLMZ3	6.2000	5.9950	0.5600	1.3628		A
COLMZ4	5.8000	5.6890	0.4700	1.3035		A
COLMZ5	7.6000	7.3892	0.5800	1.5709		B
COLMZ6	4.4000	4.2984	0.5600	1.1999		A
Zhanjiang	7.67	7.4917	0.5633 ± 0.0433 a	1.4942	0.252 ± 0.063	
COLZJ1	7.8000	7.6984	0.5600	1.4313		B
COLZJ2	9.0000	8.7688	0.4900	1.7971		B
COLZJ3	6.2000	6.0078	0.6400	1.2541		A
Beihai	6.3	6.1220	0.4117 ± 0.0717abc	1.2887	0.290 ± 0.053	
COLBH1	5.8000	5.6522	0.4833	1.2207		A
COLBH2	6.8000	6.5918	0.3400	1.3568		A
Guangzhou	6.4	6.2252	0.5660 ± 0.0337a	1.3219	0.387 ± 0.072	
COLGZ1	5.2000	5.0322	0.4400	1.0368		B
COLGZ2	6.4000	6.2922	0.6300	1.4941		D
COLGZ3	7.6000	7.3692	0.5200	1.4756		A
COLGZ4	7.0000	6.7876	0.6400	1.4242		A
COLGZ5	5.8000	5.6446	0.6000	1.1790		A
Naozhou	6.26	5.7661	0.3846 ± 0.0337 bc	1.3279	0.065 ± 0.014	
COLNZ1	5.8000	5.6614	0.4337	1.2649		C
COLNZ2	7.6000	6.2368	0.3200	1.4241		A, C
COLNZ3	5.4000	5.4000	0.4000	1.2947		A, C
Weizhou	5.4	5.3115	0.3850 ± 0.2250 abc	1.1360	0.119 ± 0.044	
COLWZ1	5.2000	5.0952	0.6100	1.2429		G
COLWZ2	5.6000	5.5278	0.1600	1.0290		C
Hebao	6.6667	6.4825	0.5000 ± 0.0346 abc	1.4219	0.321 ± 0.046	
COLHBD1	6.6000	6.4078	0.4400	1.3787		A
COLHBD2	5.6000	5.4504	0.5600	1.1525		B
COLHBD3	7.8000	7.5892	0.5000	1.7346		C
Dong'ao	6.95	6.7381	0.5643 ± 0.0378a	1.4695	0.298 ± 0.033	
COLDA1	6.8000	6.6640	0.4900	1.4488		A
COLDA2	6.8000	6.5570	0.6663	1.4914		A
COLDA3	7.8000	7.5106	0.5300	1.5317		A
COLDA4	6.4000	6.2206	0.5711	1.4059		A
Shangchuan	5.3	4.8615	0.3600 ± 0.0300c	1.0357	0.410 ± 0.033	
COLSCD1	4.8000	4.7336	0.3900	1.1551		A
COLSCD2	5.8000	4.9894	0.3300	0.9162		A, F

“A” represents the mean number of alleles per locus. AR and HO are the mean allelic richness and observed heterozygosity, respectively. *I* indicates Shannon's index. *r* is the relatedness coefficient for worker nestmates within populations. Haplotype was estimated by DnaSP. Means of observed heterozygosity of all populations were compared using Kruskal–Wallis test, and if the differences were significant, multiple comparisons of means were performed with the Mann–Whitney test. Data with the same letter indicate no significant difference at a level of .05.

### 3.2 Testing for Hardy–Weinberg equilibrium and linkage disequilibrium

Four of the six microsatellite loci exhibited significant deviations from Hardy–Weinberg equilibrium (see Table 1). Across all populations, the test for linkage disequilibrium showed no significant deviation in any of the fifteen pairwise tests (*p *>* *.05). Therefore, we considered all loci as independent. Null alleles were estimated by FreeNA software, and their frequency varied from 0 to 0.45. Locus 364 had the highest null allele frequency (0.45), and it exceeded 0.3 in most of the populations. Locus 364 was dropped from further analysis.

### 3.3 Genetic diversity of populations

**Figure 2:**
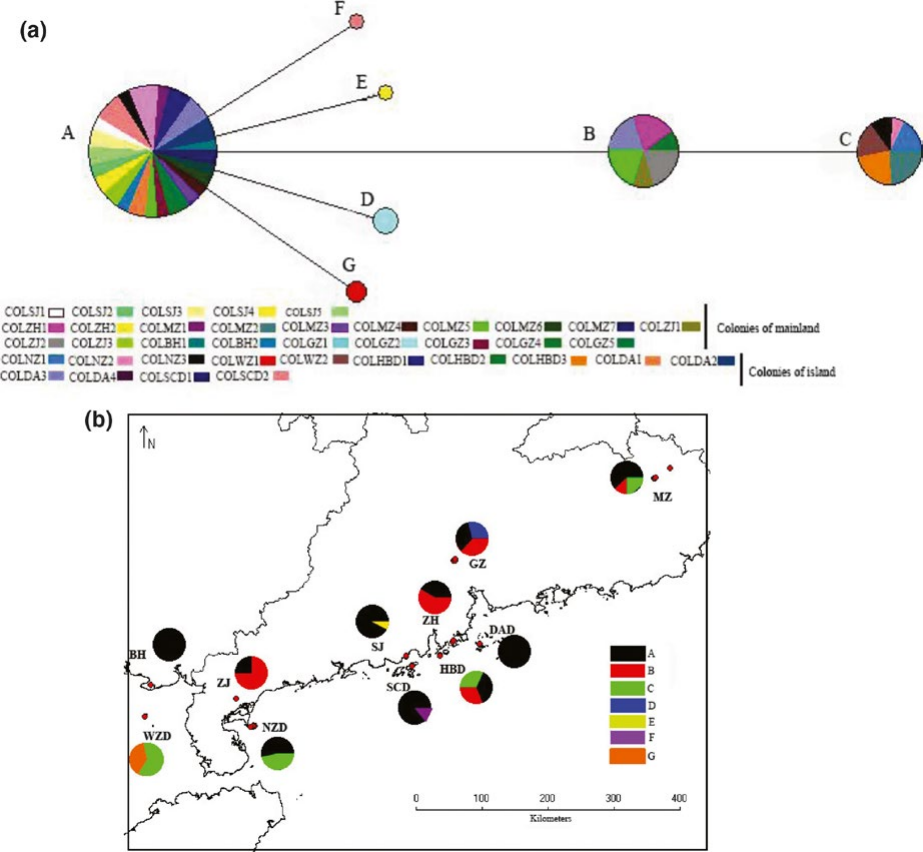
(a) The distribution of haplotype network of colonies from mainland and island populations. One circle means one haplotype; different colors stand for different colonies; circle size represents the number of the specific haplotype; A, B, C, D, E, F, and G represent haplotype name. (b) The geographic distribution of the haplotypes of mainland and island populations. Different colors stand for different haplotypes. Arcuate area represents the number of haplotypes

Overall, 778 individuals were successfully genotyped at six microsatellite loci. The results showed that the mainland populations had higher average numbers of alleles (A) and allelic richness (AR) than the island populations (Table 1), but there was no significant difference (A: *t* = 0.323, *df* = 8, *p *=* *.755; AR: *t* = 0.263, *df* = 8, *p *=* *.799). In addition, we also analyzed the genetic diversity of each colony using microsatellite and mitochondrial DNA data. The number of alleles for each colony ranged from 4.4 to 9.0, and the allelic richness varied from 4.3 to 8.8 (see Table 2). Range of observed heterozygosity among all colonies was from 0.33 (COLSCD2) to 0.67 (COLDAD2). We found that Shannon's index varied from 0.92 (COLSCD2) to 1.79 (COLZJ2), indicating that all populations and colonies exhibited a modest level of genetic diversity. At the population level, the number of alleles in all populations varied from 5.30 (SJ) to 7.67 (ZJ), allelic richness ranged from 4.8615 (SCD) to 7.49 (ZJ), and the observed heterozygosity ranged from 0.36 (SCD) to 0.57 (GZ). The results indicated that the SCD populations exhibited the lowest genetic variation. The average observed heterozygosity of the SCD points was slightly lower than that of several mainland populations (Kruskal–Wallis test*,* χ^2^ = 19.24, *df* = 10, *p *=* *.037). Therefore, with the exception of the SJ and BH populations, the genetic diversity of the SCD population was significantly lower than that of the mainland populations (*p *<* *.05). Regarding the mitochondrial DNA data (Table 2), we obtained 111 complete sequences, and the length of sequences was 610 bp. There was only a single haplotype in the DAD, BH, and SCD samples. The mainland populations had five haplotypes (A, B, C, D, and E), and the island populations had four haplotypes (A, B, C, F, and G). However, we did not find more than one haplotype in a colony. We then constructed haplotype network (Figure 2a) and the geographic distribution of haplotypes for all colonies from the mainland and island populations (Figure 2b). We found that haplotypes D, E, F, and G all specifically distributed into a single mainland and island colony, respectively. And the distribution of all haplotypes from the mainland and island populations has no linked geographically (Table 2, Figure 2b).

### 3.4 Genetic structure of populations

**Figure 3:**
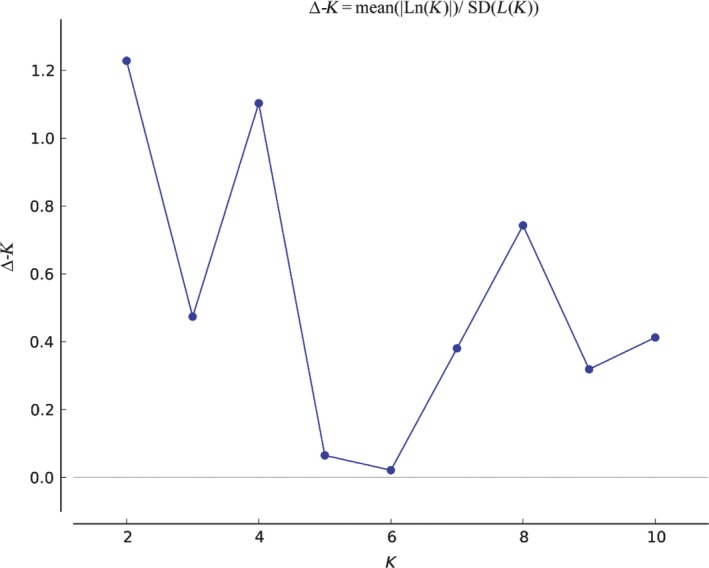
Delta‐*K* values based on 10 runs of *K* ranging from 1 to 11 using STRUCTURE

**Figure 4:**
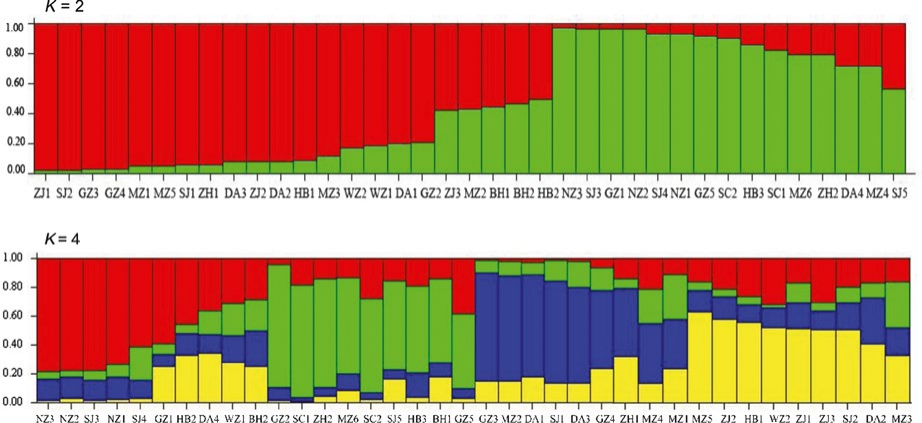
Colony genetic structure of *Tapinoma melanocephalum* based on genotypes for *K* = 2 and *K* = 4 using the program STRUCTURE. Each individual is represented by a vertical bar. The same color stands for individuals that are assigned to the same cluster

The genetic clustering of the *T. melanocephalum* populations by Bayesian clustering analysis was grouped into two branches (*K* = 2 and 4; Figure S1) by STRUCTURE using the delta‐*K* method, with 20 runs for *K* ranging from 1 to 11 (Figure 3). Interestingly, the two genetic clusters showed no relationship with the geographic location of colonies, and individuals from one site were assigned to different genetic clusters. Most of the colonies of the islands were mixed with colonies from the mainland. Approximately 50% membership of BH2 and HBD2 colonies were not uniquely assigned to either of the two genetic clusters (Figure 4). In addition, when K increased to 4, the population structure from Naozhou Island and Shangchuan Island showed more clearly approximately >75% assignment probability. Individuals from other colonies were clustered into different genetic clusters and mixed with colonies from mainland and island areas (Figure 4).

The relatedness *R* among workers within colonies over five loci varied among populations, ranging from 0.331 to 0.373, with an average of 0.337 ± 0.07, while pairwise comparisons between colonies of each population showed that relatedness varied from 0.065 to 0.410 (mean *R* = 0.355 ± 0.035) (see Table 2). The average genetic relatedness between the two colonies of the SCD population was 0.410, which is greater than zero. The higher relatedness between the SCD colonies is consistent with their lower allelic richness compared with the other more genetically diverse populations. As explained by Boomsma, Fjerdingstad, and Frydenberg (1999), relatedness among workers from the same colony headed by a once‐mated queen will average 0.75. In this study, all colonies had much lower relatedness values and were considered polygyne, with all queens probably descending from a single foundress based on the mtDNA data.

**Figure 5:**
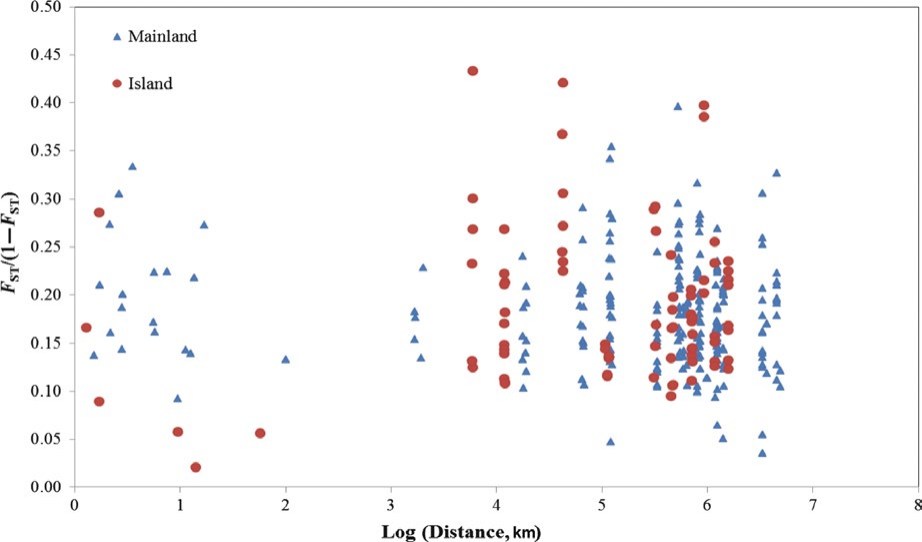
Isolation by distance (IBD) in *Tapinoma melanocephalum*. Dots indicate pairwise colony comparisons between the islands and the mainland. Circles show comparisons among 23 colonies from the mainland, and triangles show comparison for 14 colonies from the islands

We also analyzed genetic differentiation at the population level (Table 3). Among the mainland populations, some paired populations showed no differentiation (*F*_ST_ < 0). However, comparing the SCD population with most of the mainland populations showed high levels of genetic differentiation (range of *F*_ST_ = 0.1129 to 0.325), except for the nearby SJ population. Two colonies from BH and WZD were grouped into one cluster and showed no differentiation (*F*_ST_ < 0), but ZJ paired with NZD, with a similar geographic distance, exhibiting a high degree of differentiation (*F*_ST_ = 0.1721), which grouped into different clusters. The colonies from ZH, DAD, and HBD were mixed, suggesting a shared ancestry or migration between the islands and mainland due to human activity or species dispersal. When evaluating the correlation between the pairwise *F*_ST_/(1 − *F*_ST_) and the geographic distance with all colonies of the island or mainland populations, no significant correlation between genetic differentiation and geographic distance was observed (mainland: *r* = −1.00, *p *=* *.126; island: *r* = 0.117, *p *=* *.296; Figure 5).

**Table 3:** Pairwise *F*_ST_ values using five microsatellite loci

Population	SJ	ZH	MZ	ZJ	BH	GZ	NZD	WZD	HBD	DAD
ZH	0.0247									
MZ	−0.0581	0.0754								
ZJ	0.0201	0.118	0.0931							
BH	−0.0811	−0.125	−0.0221	0.0943						
GZ	−0.0509	0.0337	0.0033	0.1115	−0.1179					
NZD	0.0647	0.2376	0.1604	0.1721	0.1685	0.1086				
WZD	−0.0406	−0.0746	0.0658	−0.1207	−0.1111	−0.0052	0.0981			
HBD	−0.044	0.0811	0.0068	0.0377	−0.0185	0.0504	0.1331	−0.1037		
DAD	−0.0628	0.0415	−0.0173	0.0235	−0.0827	−0.0478	0.1556	−0.0138	0.0272	
SCD	0.0007	0.325	0.1129	0.2449	0.2051	0.1804	0.272	0.1842	0.1027	0.1441

### 3.5 Molecular variance

Hierarchical *F‐statistics* were estimated for all colonies. Three levels of hierarchical AMOVA showed that genetic differentiation was higher within colonies (*F*_IND‐COLONY_ = 0.44133, *p *<* *.001) than within the populations (*F*_REGION‐TOTAL_ = 0.20965, *p *<* *.001) and within region (*F*_COLONY‐REGION_ = 0.26028, *p *<* *.001; Table 4). For the mainland and island regions, the degree of genetic differentiation was unevenly distributed within the hierarchy. For locus 592, genetic differentiation showed the highest level between populations, not within colonies. Genetic variation in all populations revealed negative *F*_COLONY‐REGION_ values, which was considered to represent a heterozygosity excess between colonies.

**Table 4:** Population differentiation estimated by *F*‐statistics for 11 geographic regions

Locus	Total populations			Mainland (23 colonies)			Island (14 colonies)		
	F_IND‐COLONY_	F_COLONY‐REGION_	F_REGION‐TOTAL_	F_IND‐COLONY_	F_COLONY‐REGION_	F_REGION‐TOTAL_	F_IND‐COLONY_	F_COLONY‐REGION_	F_REGION‐TOTAL_
Locus 434	0.43047	0.25812	0.21420	0.39968	0.25635	0.19274	0.47890	0.26144	0.29444
Locus 592	0.09325	−0.12799	0.16977	0.04769	−0.16277	0.18099	0.15466	−0.07762	0.21555
Locus 911	0.69012	0.57276	0.21617	0.65949	0.54737	0.24772	0.73267	0.61491	0.30580
Locus 1178	0.36269	0.16192	0.20135	0.28522	0.02921	0.26372	0.46904	0.33734	0.19875
Locus 1315	0.4462	0.52937	0.2533	0.64849	0.50185	0.29437	0.67821	0.56725	0.25641
All	0.44133***	0.26028***	0.20965***	0.40188***	0.21876***	0.23440***	0.49386***	0.32243***	0.25301***

Asterisks represent significant differences from zero: *p < .05; **p < .01, and ***p < .001.

### 3.6 Bottleneck effect

We examined signatures of a genetic bottleneck using BOTTLENECK, with the two‐mutation model. The results revealed a significant heterozygosity excess (*p *<* *.05) in 11 geographic populations, except for the BH, NZD, and SCD populations under the Wilcoxon signed‐rank test of the infinite allele model (Table 5).

**Table 5:** Results of bottleneck testing in 11 geographic populations under Wilcoxon test and sign test of two‐mutation models

Population	IAM		SMM	
	Wilcoxon test	Sign test	Wilcoxon test	Sign test
SJ	0.01563*	0.0851	1	0.01119
ZH	0.04688*	0.33793	0.89063	0.34047
MZ	0.01563*	0.07692	1	0.01183
ZJ	0.04688*	0.35264	1	0.01349
BH	0.3125	0.32616	1	0.01241
GZ	0.03125*	0.35684	0.98438	0.0885
NZD	0.40625	0.33165	1	0.01318
WZD	0.03125*	0.29993	1	0.01172
HBD	0.01563*	0.08034	0.98438	0.10423
DAD	0.01563*	0.0836	1	0.01203
SCD	0.92188	0.33973	1	0.01183

There was no significant heterozygosity excess in any of the populations determined by the sign test performed with the infinite allele model (*p *>* *.05). The stepwise mutation model (SMM) showed no significant heterozygosity excess in any population. However, the sign test with the stepwise mutation model exhibited significant heterozygosity excess in all populations except GZ, ZH, and HBD populations (*p *<* *.05).

Our results suggest that the populations of SJ, ZJ, MZ, WZD, and DAD experienced a recent genetic bottleneck under the Wilcoxon test and sign test of two‐mutation models (Table 5).

## 4 DISCUSSION

### 4.1 Genetic diversity of populations

Study of genetic diversity and potential for rapid evolution is helpful for understanding colonization dynamics and spread of invasive social insects (Sakai et al., 2001). Geographic characteristics can affect dispersal range and gene flow of invasive species and further influence genetic diversity of introduced populations (Barrett & Husband, 1990; Moody & Mack, 1988). For islands, a natural barrier has the potential to influence species diversity or population size (Jensen et al., 2013). In our study, the genetic diversity of the mainland populations of *T. melanocephalum* was slightly higher than that of the island populations, but there were no significant differences. However, colonies in the SCD population had lower allelic richness and number of alleles than mainland colonies; the observed heterozygosity of the SCD population was dramatically lower than that of several mainland populations. Multiple introductions of invasive populations could promote higher genetic diversity of alien species compared with a single introduction (Sakai et al., 2001; Wilson, Naish, & Boulding, 1999). In our study, microsatellite data showed that the genetic diversity of island populations was close to that of the mainland populations. This was also supported by mitochondrial DNA data. One possible reason is that populations from the mainland and the islands are multiply introduced with movement of human beings and frequent commercial trade.

However, invasive species are usually associated with population bottlenecks in the introduced regions and reduction in diversity may occur due to genetic drift during colonization (Barrett & Husband, 1990). Low levels of heterozygosity have been taken as evidence of bottlenecks in populations in the recent past (less than four *N*_*e*_ generations ago) (Luikart & Cornuet, 1998). Interestingly, Argentine ants in the southeastern United States (introduced populations) experienced bottlenecks and exhibit higher levels of genetic diversity than other introduced populations (Buczkowski, Vargo, & Silverman, 2004). Some populations may display lower genetic diversity while no bottleneck effect is detected. This may occur if the population has been present for long periods of time during which they recover from the signatures of a bottleneck (Green, 1990). In our study, most island populations of *T. melanocephalum* showed similar allele richness with that of the mainland populations, and three island populations experienced no detectable bottleneck.

### 4.2 Population genetic structure

Using the STRUCTURE program, we obtained two genetic clusters for all *T. melanocephalum* populations, which did not correspond to distinct geographic locations and admixtures of island and mainland colonies. *T. melanocephalum* has been described as an intranidal mating species and was shown to form unicolonial populations by colony budding (Hölldobler & Wilson, 1977). Gene flow has shown to be limited between different colonies or supercolonies because of intranidal mating (Espadaler & Rey, 2001; Helanterä, Strassmann, Carrillo, & Queller, 2009). STRUCTURE analysis showed some colonies could not be assigned to either of the two clusters. This result suggests that the clusters are not completely distinct. It may be attributed to long‐distance dispersal of the ghost ant that frequently occurs due to human activities (Seifert, 2010). Therefore, newly introduced populations may mix with earlier arrivals to form a single more genetically diverse unicolonial society, thus promoting gene flow at large scales among island and mainland populations. On the other hand, based on our results showing each colony had a single mtDNA haplotype, it appears that each of our study colonies descended from a single queen. It also suggests colonies do not fuse the way Argentine ant colonies do (Thomas et al., 2010). Notably, colonies of most of populations we tested were not uniquely assigned to either of the two genetic clusters by STRUCTURE analysis using microsatellite data. In addition, most workers in our study were heterozygous at microsatellite loci. Taken together, this may indicate a special breeding structure in this species in which mating occurs in or near the nest and a majority of males come from external nests. Obviously, the reproductive habits of this species in China feature need further study.

Among the paired mainland populations, there was no differentiation. However, when the SCD populations were paired with most of the mainland populations, we found a high level of genetic differentiation, and *F*_ST_s ranged from 0.0007 to 0.325. In particular, the SCD populations and the ZH populations may have different origins leading to the observed strong genetic differentiation. This is supported by one of the ZH colonies, ZH1, having a different mtDNA haplotype than the SCD colonies. There was no correlation between the genetic differentiation and geographic distance among all colonies, which suggests that gene flow among colonies is not related to distance, and isolation by sea has had minimal influence on gene flow. Five islands were separated by 16–50 km from the nearest mainland.

Hierarchical AMOVA suggests that the genetic divergence of *T. melanocephalum* was mainly due to differences within colonies instead of between colonies in regions or sampling regions. Due to founder events, colonies achieved rapid differentiation. The changes in allele frequency were affected by differential survival of individuals during the initial bottleneck (Chakraborty & Nei, 1977; Tarr, Conant, & Fleischer, 1998).

Our results indicated low relatedness within all individuals of colonies. But, in the SCD population, colonies exhibited the highest *r* value (0.410) and the lowest allelic richness; these colonies all clustered together. Similar to a previous report, invasive species in introduced regions tend to form supercolonies or colonies (Holway et al., 2002) with reduced genetic variability and increased nestmate recognition (Payne, Tillberg, & Suarez, 2004; Tsutsui et al., 2000). This may also be attributable to the possibility that the SCD population was derived from only one introduction. Strong hurricanes and fierce floods may also affect the population distribution and expansion and could also lead to genetic loss. In addition, mtDNA data were uniform within a colony, suggesting that each colony came from a single matriline.

Many reports of invasive species in different geographic regions suggest that social behavior is related to colony breeding structure. In its native range, the Argentine ant forms small supercolonies that are effectively unicolonial by intranidal mating and kin recognition (Pedersen, Krieger, Vogel, Giraud, & Keller, 2006). Intranidal mating has also been reported in *Monomorium pharaonis* (Wilson, 1971) and *Lasius sakagamii* ant species (Yamauchi, Kinomura, & Miyake, 1981), as well as *T. melanocephalum*, the Argentine ant, and *Anoplolepis gracilipes* (Bustos, Cherix, Lachaud, & Fourcassié, 1998; Drescher, Blüthgen, Schmitt, Bühler, & Feldhaar, 2010). A previous report revealed a negative correlation between relatedness and aggression in *Anoplolepis gracilipes*, which suggested kinship as one factor causing the formation of a supercolony. Kinship also facilitates colonization (Drescher, Blüthgen, & Feldhaar, 2007). Supercolonies of *A. gracilipes* undergo random mating; intranidal mating system in this ant can be observed due to genetic cohesion in these two supercolonies (Thomas et al., 2010). In the introduced range, the invasive red imported fire ant exhibits a higher degree of polygyny than in native regions (Ross & Keller, 1995). Polygynous colonies in native areas have fewer queens who are close relatives compared to the many unrelated queens found in introduced populations (Ross, Vargo, & Keller, 1996), leading to the observed high queen numbers and decreased relatedness for introduced ranges.

Closely related colony members in polygynous nests may arise from inbreeding, reproductive dominance of one queen, and/or high relatedness among queens (Giraud, Blatrix, Poteaux, Solignac, & Jaisson, 2001). Nestmate queens in polygynous species are generally related, but to a degree that varies greatly between species, some species having nestmate queens that are almost unrelated (Keller, 1995). Therefore, the queen number and relatedness among nestmate queens of *T. melanocephalum* should also be a focus of future studies as well the relationship between colonies in China and those in other parts of the world.

## CONFLICT OF INTEREST

None declared.

## References

[ece34065-bib-0001] Allee, W. C. , Park, O. , Emerson, A. E. , Park, T. , & Schmidt, K. P. (1949). Principles of animal ecology. Philadelphia: WB Saunders Co. Ltd.

[ece34065-bib-0002] Antonovics, J. (1976). The nature of limits to natural selection. Annals of the Missouri Botanical Garden, 63, 224–247. https://doi.org/10.2307/2395303

[ece34065-bib-0003] Barnett, R. , Cucchi, T. , Martinkova, N. , Struchan, R. , Pascal, M. , Pascal, M. , … Ho, S. (2013). Divergent evolutionary processes associated with colonization of offshore islands. Molecular Ecology, 22, 5205–5220. 23998800 10.1111/mec.12462 PMC4159590

[ece34065-bib-0004] Barrett, S. C. H. , & Husband, B. C. (1990). Genetics of plant migration and colonization In Brown A. H. D., Clegg M. T., Kahler A. L., & Weir B. S. (Eds.), Plant population genetics, breeding, and genetic resources (pp. 254–277). Sunderland, MA: Sinauer.

[ece34065-bib-0005] Beardmore, J. , Mair, G. , & Lewis, R. (1997). Biodiversity in aquatic systems in relation to aquaculture. Aquaculture Research, 28, 829–839. https://doi.org/10.1111/j.1365-2109.1997.tb01007.x

[ece34065-bib-0006] Boomsma, J. J. , Fjerdingstad, E. J. , & Frydenberg, J. (1999). Multiple paternity, relatedness and genetic diversity in *Acromyrmex* leaf‐cutter ants. Proceedings of the Royal Society B Biological Sciences, 266, 249 https://doi.org/10.1098/rspb.1999.0629

[ece34065-bib-0007] Bourke, A. F. , & Franks, N. R. (1995). Social evolution in ants. Princeton, NJ: Princeton University Press.

[ece34065-bib-0008] Buczkowski, G. , Vargo, E. L. , & Silverman, J. (2004). The diminutive supercolony: The Argentine ants of the southeastern United States. Molecular Ecology, 13, 2235–2242. https://doi.org/10.1111/j.1365-294X.2004.02261.x 15245397 10.1111/j.1365-294X.2004.02261.x

[ece34065-bib-0009] Bustos, X. , Cherix, D. , Lachaud, J. , & Fourcassié, V. (1998) Contribution to the biology of Tapinoma melanocephalum (Fabricius)(Hymenoptera: Formicidae). Actes des Colloques Insectes Sociaux, vol. 11: Compte rendu colloque annuel, Créteil, France, 3–5 Septembre 1997, pp. 95–101.

[ece34065-bib-0010] Chakraborty, R. , & Nei, M. (1977). Bottleneck effects on average heterozygosity and genetic distance with the stepwise mutation model. Evolution, 31, 347–356. https://doi.org/10.1111/j.1558-5646.1977.tb01017.x 28563234 10.1111/j.1558-5646.1977.tb01017.x

[ece34065-bib-0011] Chapuis, M.‐P. , & Estoup, A. (2007). Microsatellite null alleles and estimation of population differentiation. Molecular Biology and Evolution, 24, 621–631. https://doi.org/10.1093/molbev/msl191 17150975 10.1093/molbev/msl191

[ece34065-bib-0012] Chiotis, M. , Jermiin, L. S. , & Crozier, R. H. (2000). A molecular framework for the phylogeny of the ant subfamily Dolichoderinae. Molecular Phylogenetics and Evolution, 17, 108–116. https://doi.org/10.1006/mpev.2000.0821 11020309 10.1006/mpev.2000.0821

[ece34065-bib-0013] Colwell, R. K. , Mao, C. X. , & Chang, J. (2004). Interpolating, extrapolating, and comparing incidence‐based species accumulation curves. Ecology, 85, 2717–2727. https://doi.org/10.1890/03-0557

[ece34065-bib-0014] Corin, S. , Abbott, K. , Ritchie, P. , & Lester, P. (2007). Large scale unicoloniality: The population and colony structure of the invasive Argentine ant (*Linepithema humile*) in New Zealand. Insectes Sociaux, 54, 275–282. https://doi.org/10.1007/s00040-007-0942-9

[ece34065-bib-0015] Deheer, C. J. , & Vargo, E. L. (2004). Colony genetic organization and colony fusion in the termite *Reticulitermes flavipes* as revealed by foraging patterns over time and space. Molecular Ecology, 13, 431–441. https://doi.org/10.1046/j.1365-294X.2003.2065.x 14717897 10.1046/j.1365-294x.2003.2065.x

[ece34065-bib-0016] Dempster, A. P. , Laird, N. M. , & Rubin, D. B. (1977). Maximum likelihood from incomplete data via the EM algorithm. Journal of the Royal Statistical Society Series B (Methodological), 30, 1–38.

[ece34065-bib-0017] Drescher, J. , Blüthgen, N. , & Feldhaar, H. (2007). Population structure and intraspecific aggression in the invasive ant species *Anoplolepis gracilipes* in Malaysian Borneo. Molecular Ecology, 16, 1453–1465. https://doi.org/10.1111/j.1365-294X.2007.03260.x 17391269 10.1111/j.1365-294X.2007.03260.x

[ece34065-bib-0018] Drescher, J. , Blüthgen, N. , Schmitt, T. , Bühler, J. , & Feldhaar, H. (2010). Societies drifting apart? Behavioural, genetic and chemical differentiation between supercolonies in the yellow crazy ant *Anoplolepis gracilipes* . PLoS ONE, 5, e13581 https://doi.org/10.1371/journal.pone.0013581 21042578 10.1371/journal.pone.0013581 PMC2962633

[ece34065-bib-0019] Dronnet, S. , Chapuisat, M. , Vargo, E. L. , & Bagnères, A. G. (2005). Genetic analysis of the breeding system of an invasive subterranean termite, *Reticulitermes santonensis*, in urban and natural habitats. Molecular Ecology, 14, 1311–1320. https://doi.org/10.1111/j.1365-294X.2005.02508.x 15813772 10.1111/j.1365-294X.2005.02508.x

[ece34065-bib-0020] Duffie, C. V. , Glenn, T. C. , Vargas, F. H. , & Parker, P. G. (2009). Genetic structure within and between island populations of the flightless cormorant (*Phalacrocorax harrisi*). Molecular Ecology, 18, 2103–2111. https://doi.org/10.1111/j.1365-294X.2009.04179.x 19635072 10.1111/j.1365-294X.2009.04179.x

[ece34065-bib-0021] Espadaler, X. , & Espejo, F. (2002). *Tapinoma melanocephalum* (Fabricius, 1793), a new exotic ant in Spain (Hymenoptera, Formicidae). Orsis: organismes i sistemes, 17, 101–104.

[ece34065-bib-0022] Espadaler, X. , & Rey, S. (2001). Biological constraints and colony founding in the polygynous invasive ant *Lasius neglectus* (Hymenoptera, Formicidae). Insectes Sociaux, 48, 159–164. https://doi.org/10.1007/PL00001760

[ece34065-bib-0023] Evanno, G. , Regnaut, S. , & Goudet, J. (2005). Detecting the number of clusters of individuals using the software STRUCTURE: A simulation study. Molecular Ecology, 14, 2611–2620. https://doi.org/10.1111/j.1365-294X.2005.02553.x 15969739 10.1111/j.1365-294X.2005.02553.x

[ece34065-bib-0024] Fowler, H. , Bernardi, J. , Delabie, J. , Forti, L. , & Pereira‐da‐Silva, V. (1990). Major ant problems of South America (pp. 3–14). Boulder, CO: Applied Myrmecology–a world perspective. Westview Press.

[ece34065-bib-0025] Frankel, O. , & Soulé, M. E. (1981). Conservation and evolution. Cambridge, UK: CUP Archive.

[ece34065-bib-0026] Frankham, R. , Briscoe, D. A. , & Ballou, J. D. (2002). Introduction to conservation genetics. Cambridge, UK: Cambridge University Press https://doi.org/10.1017/CBO9780511808999

[ece34065-bib-0027] Giraud, T. , Blatrix, R. , Poteaux, C. , Solignac, M. , & Jaisson, P. (2001). High genetic relatedness among nestmate queens in the polygynous ponerine ant *Gnamptogenys striatula* in Brazil. Behavioral Ecology and Sociobiology, 49, 128–134. https://doi.org/10.1007/s002650000284

[ece34065-bib-0028] Giraud, T. , Pedersen, J. S. , & Keller, L. (2002). Evolution of supercolonies: The Argentine ants of southern Europe. Proceedings of the National Academy of Sciences, 99, 6075–6079. https://doi.org/10.1073/pnas.092694199 10.1073/pnas.092694199 PMC122904 11959924

[ece34065-bib-0029] Goudet, J. (1995). FSTAT (version 1.2): A computer program to calculate F‐statistics. Journal of Heredity, 86, 485–486. https://doi.org/10.1093/oxfordjournals.jhered.a111627

[ece34065-bib-0030] Green, O. (1990). Entomologist sets new record at Mt Smart for *Iridomyrmex humilis* established in New Zealand. Weta, 13, 14–16.

[ece34065-bib-0031] Hebert, P. D. , Ratnasingham, S. , & de Waard, J. R. (2003). Barcoding animal life: Cytochrome c oxidase subunit 1 divergences among closely related species. Proceedings of the Royal Society of London B: Biological Sciences, 270, S96–S99. https://doi.org/10.1098/rsbl.2003.0025 10.1098/rsbl.2003.0025 PMC1698023 12952648

[ece34065-bib-0032] Hedrick, P. W. , & Kalinowski, S. T. (2000). Inbreeding depression in conservation biology. Annual Review of Ecology and Systematics, 31, 139–162. https://doi.org/10.1146/annurev.ecolsys.31.1.139

[ece34065-bib-0033] Helanterä, H. , Strassmann, J. E. , Carrillo, J. , & Queller, D. C. (2009). Unicolonial ants: Where do they come from, what are they and where are they going? Trends in Ecology & Evolution, 24, 341–349. https://doi.org/10.1016/j.tree.2009.01.013 19328589 10.1016/j.tree.2009.01.013

[ece34065-bib-0034] Hölldobler, B. , & Wilson, E. O. (1977). The number of queens: An important trait in ant evolution. Naturwissenschaften, 64, 8–15. https://doi.org/10.1007/BF00439886

[ece34065-bib-0035] Holway, D. A. , Lach, L. , Suarez, A. V. , Tsutsui, N. D. , & Case, T. J. (2002). The causes and consequences of ant invasions. Annual Review of Ecology and Systematics, 33, 181–233. https://doi.org/10.1146/annurev.ecolsys.33.010802.150444

[ece34065-bib-0036] Holway, D. A. , & Suarez, A. V. (2004). Colony‐structure variation and interspecific competitive ability in the invasive Argentine ant. Oecologia, 138, 216–222. https://doi.org/10.1007/s00442-003-1414-1 14566557 10.1007/s00442-003-1414-1

[ece34065-bib-0037] Jensen, H. , Moe, R. , Hagen, I. J. , Holand, A. M. , Kekkonen, J. , Tufto, J. , & Sæther, B. E. (2013). Genetic variation and structure of house sparrow populations: Is there an island effect? Molecular Ecology, 22, 1792–1805. https://doi.org/10.1111/mec.12226 23379682 10.1111/mec.12226

[ece34065-bib-0038] Keller, L. (1995). Social life: The paradox of multiple‐queen colonies. Trends in Ecology & Evolution, 10, 355–360. https://doi.org/10.1016/S0169-5347(00)89133-8 21237068 10.1016/s0169-5347(00)89133-8

[ece34065-bib-0039] Linhart, Y. B. , & Grant, M. C. (1996). Evolutionary significance of local genetic differentiation in plants. Annual Review of Ecology and Systematics, 27, 237–277. https://doi.org/10.1146/annurev.ecolsys.27.1.237

[ece34065-bib-0040] Losos, J. B. , & Ricklefs, R. E. (2009a). The theory of island biogeography revisited. Princeton, NJ: Princeton University Press https://doi.org/10.1515/9781400831920

[ece34065-bib-0041] Losos, J. B. , & Ricklefs, R. E. (2009b). Adaptation and diversification on islands. Nature, 457, 830–836. https://doi.org/10.1038/nature07893 19212401 10.1038/nature07893

[ece34065-bib-0042] Luikart, G. , & Cornuet, J. M. (1998). Empirical evaluation of a test for identifying recently bottlenecked populations from allele frequency data. Conservation Biology, 12, 228–237. https://doi.org/10.1046/j.1523-1739.1998.96388.x

[ece34065-bib-0043] Moody, M. E. , & Mack, R. N. (1988). Controlling the spread of plant invasions: The importance of nascent foci. Journal of Applied Ecology, 25, 1009–1021. https://doi.org/10.2307/2403762

[ece34065-bib-0044] Morley, M. , Molony, C. M. , Weber, T. M. , Devlin, J. L. , Ewens, K. G. , Spielman, R. S. , & Cheung, V. G. (2004). Genetic analysis of genome‐wide variation in human gene expression. Nature, 430, 743–747. https://doi.org/10.1038/nature02797 15269782 10.1038/nature02797 PMC2966974

[ece34065-bib-0045] Nickerson, J. , Bloomcamp, C. , & Fasulo, T.R. (2004) Ghost Ant, Tapinoma melanocephalum (Fabricius)(Insecta: Hymenoptera: Formicidae). Citeseer.

[ece34065-bib-0046] Parker, I. M. , Rodriguez, J. , & Loik, M. E. (2003). An evolutionary approach to understanding the biology of invasions: Local adaptation and general‐purpose genotypes in the weed *Verbascum thapsus* . Conservation Biology, 17, 59–72. https://doi.org/10.1046/j.1523-1739.2003.02019.x

[ece34065-bib-0047] Payne, C. M. , Tillberg, C. V. , & Suarez, A. V. (2004). Recognition systems and biological invasions In Annales Zoologici Fennici (pp. 843–858). 41: Finnish Zoological and Botanical Publishing Board.

[ece34065-bib-0048] Peakall, R. , & Smouse, P. E. (2006). GENALEX 6: Genetic analysis in Excel. Population genetic software for teaching and research. Molecular Ecology Notes, 6, 288–295. https://doi.org/10.1111/j.1471-8286.2005.01155.x 10.1093/bioinformatics/bts460 PMC3463245 22820204

[ece34065-bib-0049] Pedersen, J. S. , Krieger, M. J. , Vogel, V. , Giraud, T. , & Keller, L. (2006). Native supercolonies of unrelated individuals in the invasive Argentine ant. Evolution, 60, 782–791. https://doi.org/10.1111/j.0014-3820.2006.tb01156.x 16739459

[ece34065-bib-0050] Pimm, S. L. , Diamond, J. , Reed, T. M. , Russell, G. J. , & Verner, J. (1993). Times to extinction for small populations of large birds. Proceedings of the National Academy of Sciences, 90, 10871–10875. https://doi.org/10.1073/pnas.90.22.10871 10.1073/pnas.90.22.10871 PMC47880 11607439

[ece34065-bib-0051] Piry, S. , Luikart, G. , & Cornuet, J.‐M. (1999). BOTTLENECK: A program for detecting recent effective population size reductions from allele data frequencies. Journal of Heredity, 90, 502–503. https://doi.org/10.1093/jhered/90.4.502

[ece34065-bib-0052] Pritchard, J. , Wen, X. , & Falush, D. (2010) Documentation for structure software: Version 2.2. Retrieved from: http://pritch.bsd.uchicago.edu/software/structure22/readme.pdf. Accessed 25 June 2014.

[ece34065-bib-0053] Queller, D. C. , & Goodnight, K. F. (1989). Estimating relatedness using genetic markers. Evolution, 42(2), 258–275. https://doi.org/10.1111/j.1558-5646.1989.tb04226.x 10.1111/j.1558-5646.1989.tb04226.x 28568555

[ece34065-bib-0054] Raymond, M. , & Rousset, F. (1995). GENEPOP (version 1.2): Population genetics software for exact tests and ecumenicism. Journal of Heredity, 86, 248–249. https://doi.org/10.1093/oxfordjournals.jhered.a111573

[ece34065-bib-0055] Rice, W. R. (1989). Analyzing tables of statistical tests. Evolution, 43, 223–225. https://doi.org/10.1111/j.1558-5646.1989.tb04220.x 28568501 10.1111/j.1558-5646.1989.tb04220.x

[ece34065-bib-0056] Ross, K. G. , & Keller, L. (1995). Ecology and evolution of social organization: Insights from fire ants and other highly eusocial insects. Annual Review of Ecology and Systematics, 26, 631–656. https://doi.org/10.1146/annurev.es.26.110195.003215

[ece34065-bib-0057] Ross, K. G. , Vargo, E. L. , & Keller, L. (1996). Social evolution in a new environment: The case of introduced fire ants. Proceedings of the National Academy of Sciences, 93, 3021–3025. https://doi.org/10.1073/pnas.93.7.3021 10.1073/pnas.93.7.3021 PMC39754 11607647

[ece34065-bib-0058] Rousset, F. (1997). Genetic differentiation and estimation of gene flow from F‐statistics under isolation by distance. Genetics, 145, 1219–1228. 9093870 10.1093/genetics/145.4.1219 PMC1207888

[ece34065-bib-0059] Rozas, J. , Sánchez‐DelBarrio, J. C. , Messeguer, X. , & Rozas, R. (2003). DnaSP, DNA polymorphism analyses by the coalescent and other methods. Bioinformatics, 19, 2496–2497. https://doi.org/10.1093/bioinformatics/btg359 14668244 10.1093/bioinformatics/btg359

[ece34065-bib-0060] Sakai, A. K. , Allendorf, F. W. , Holt, J. S. , Lodge, D. M. , Molofsky, J. , With, K. A. , … Ellstrand, N. C. (2001). The population biology of invasive species. Annual Review of Ecology and Systematics, 32, 305–332. https://doi.org/10.1146/annurev.ecolsys.32.081501.114037

[ece34065-bib-0061] Schneider, S. , Roessli, D. , & Excoffier, L. (2000). Arlequin: A software for population genetics data analysis. User Manual Ver, 2, 2496–2497.

[ece34065-bib-0062] Seifert, B. (2010). Intranidal mating, gyne polymorphism, polygyny, and supercoloniality as factors for sympatric and parapatric speciation in ants. Ecological Entomology, 35, 33–40. https://doi.org/10.1111/j.1365-2311.2009.01136.x

[ece34065-bib-0063] Smith, E. H. , & Whitman, R. C. (1992). Field guide to structural pests. Dunn Loring, VA: National Pest Management Association.

[ece34065-bib-0064] Tarr, C. , Conant, S. , & Fleischer, R. (1998). Founder events and variation at microsatellite loci in an insular passerine bird, the *Laysan finch* (Telespiza cantans). Molecular Ecology, 7, 719–731. https://doi.org/10.1046/j.1365-294x.1998.00385.x

[ece34065-bib-0065] Thomas, M. L. , Becker, K. , Abbott, K. , & Feldhaar, H. (2010). Supercolony mosaics: Two different invasions by the yellow crazy ant, *Anoplolepis gracilipes*, on Christmas Island, Indian Ocean. Biological Invasions, 12, 677–687. https://doi.org/10.1007/s10530-009-9473-9

[ece34065-bib-0501] Thorne, B. L. , Traniello, J. F. A. , Adams, E. S. , & Bulmer, M. (1999). Reproductive dynamics and colony structure of subterranean termites of the genus Reticulitermes (Isoptera: Rhinotermitidae): A review of the evidence from behavioral, ecological, and genetic studies. Ethology Ecology & Evolution, 11(2), 149–169.

[ece34065-bib-0066] Tsutsui, N. D. , Suarez, A. V. , Holway, D. A. , & Case, T. J. (2000). Reduced genetic variation and the success of an invasive species. Proceedings of the National Academy of Sciences, 97, 5948–5953. https://doi.org/10.1073/pnas.100110397 10.1073/pnas.100110397 PMC18539 10811892

[ece34065-bib-0067] Vargo, E. L. (2003). Hierarchical analysis of colony and population genetic structure of the eastern subterranean termite, *Reticulitermes flavipes*, using two classes of molecular markers. Evolution, 57, 2805–2818. https://doi.org/10.1111/j.0014-3820.2003.tb01522.x 14761059 10.1111/j.0014-3820.2003.tb01522.x

[ece34065-bib-0068] Vásquez, G. M. , & Silverman, J. (2008). Intraspecific aggression and colony fusion in the Argentine ant. Animal Behaviour, 75, 583–593. https://doi.org/10.1016/j.anbehav.2007.06.019

[ece34065-bib-0069] Venkataramaiah, G. , & Rehman, P. (1989). Ants associated with the mealybugs of coffee. Indian Coffee, 43, 13–14.

[ece34065-bib-0070] Waldman, B. , Frumhoff, P. C. , & Sherman, P. W. (1988). Problems of kin recognition. Trends in Ecology & Evolution, 3, 8–13. https://doi.org/10.1016/0169-5347(88)90075-4 21227051 10.1016/0169-5347(88)90075-4

[ece34065-bib-0071] Weir, B. S. , & Cockerham, C. C. (1984). Estimating *F*‐statistics for the analysis of population structure. Evolution, 38, 1358–1370. 28563791 10.1111/j.1558-5646.1984.tb05657.x

[ece34065-bib-0072] Wetterer, J. K. (2009). Worldwide spread of the ghost ant, *Tapinoma melanocephalum* (Hymenoptera: Formicidae). Myrmecological News, 12, 23–33.

[ece34065-bib-0073] Wilson, E. O. (1967). The ants of Polynesia (Hymenoptera: Formicidae). Annual Review of Ecology and Systematics, 33, 181–233.

[ece34065-bib-0074] Wilson, E. O. (1971). The insect societies. Cambridge, MA: Harvard University. Press.

[ece34065-bib-0075] Wilson, A. , Naish, K.‐A. , & Boulding, E. (1999). Multiple dispersal strategies of the invasive quagga mussel (*Dreissena bugensis*) as revealed by microsatellite analysis. Canadian Journal of Fisheries and Aquatic Sciences, 56, 2248–2261. https://doi.org/10.1139/f99-162

[ece34065-bib-0076] Yamauchi, K. , Kinomura, K. , & Miyake, S. (1981). Sociobiological studies of the polygynic ant *Lasius sakagamii* . Insectes Sociaux, 28, 279–296. https://doi.org/10.1007/BF02223629

