# Supplementary material for: Genetic diversity and colony structure of Tapinoma melanocephalum on the islands and mainland of South China
Source: Ecol Evol. 2018 May 2;8(11):5427–40. doi: 10.1002/ece3.4065 (PMC6010919; doi:10.1002/ece3.4065)
Supplement: Supplementary file 1 [file ECE3-8-5427-s001.docx]

**Genetic diversity and colony structure of** ***Tapinoma melanocephalum* on the islands and mainland of South China**

**Running title: Genetic diversity and structure of ghost ant**

Chunyan Zheng^1^, Fan Yang^1^, Ling Zeng^1^, Edward L. Vargo^2*^ and Yijuan Xu^1*^

1 South China Agricultural University, Department of Entomology, Guangzhou 510640, China

2 Texas A&M Univ, Dept Entomol, College Stn, TX 77843 USA

*Correspondence: Yijuan Xu (xuyijuan@yahoo.com) and Edward L. Vargo ([ed.vargo@tamu.edu](mailto:ed.vargo@tamu.edu))

Table S1. Collection site information.

| Populations | Collection points | Colonies identified | Sample  number | Longitude（E） | Latitude（N） |
| --- | --- | --- | --- | --- | --- |
| Mainland Total | 23 | 23 | 460 |  |  |
| BH | BH1 | COLBH1 | 20 | 109°10’47’’ | 21°28’01’’ |
|  | BH2 | COLBH2 | 20 | 109°10’55’’ | 21°27’51’’ |
| ZJ | ZJ1 | COLZJ1 | 20 | 110°21’55’’ | 21°16’40’’ |
|  | ZJ2 | COLZJ2 | 20 | 110°21’40’’ | 21°16’35’’ |
|  | ZJ3 | COLZJ3 | 20 | 110°21’32’’ | 21°16’39’’ |
| SJ | SJ1 | COLSJ1 | 20 | 112°41’56’’ | 21°51’20’’ |
|  | SJ2 | COLSJ2 | 20 | 112°41’57’’ | 21°51’22’’ |
|  | SJ3 | COLSJ3 | 20 | 112°42’00’’ | 21°51’24’’ |
|  | SJ4 | COLSJ4 | 20 | 112°42’05’’ | 21°51’43’’ |
|  | SJ5 | COLSJ5 | 20 | 112°42’03’’ | 21°52’03’’ |
| ZH | ZH1 | COLZH1 | 20 | 113°20’44’’ | 22°03’16’’ |
|  | ZH2 | COLZH2 | 20 | 113°21’42’’ | 22°04’33’’ |
| GZ | GZ1 | COLGZ1 | 20 | 113°21’58’’ | 23°09’58’’ |
|  | GZ2 | COLGZ2 | 20 | 113°21’09’’ | 23°09’54’’ |
|  | GZ3 | COLGZ3 | 20 | 113°22’52’’ | 23°09’31’’ |
|  | GZ4 | COLGZ4 | 20 | 113°22’36’’ | 23°09’23’’ |
|  | GZ5 | COLGZ5 | 20 | 113°22’42’’ | 23°11’03’’ |
| MZ | MZ1 | COLMZ1 | 20 | 116°07’26’’ | 24°16’38’’ |
|  | MZ2 | COLMZ2 | 20 | 116°07’40’’ | 24°16’54’’ |
|  | MZ3 | COLMZ3 | 20 | 116°08’33’’ | 24°17’10’’ |
|  | MZ4 | COLMZ4 | 20 | 116°08’33’’ | 24°17’08’’ |
|  | MZ5 | COLMZ5 | 20 | 116°08’34’’ | 24°17’07’’ |
|  | MZ6 | COLMZ6 | 20 | 116°20’34’’ | 24°25’10’’ |
| Island Total | 16 | 14 | 318 |  |  |
| WZD | WZD1 | COLWZD1 | 20 | 109°06’00’’ | 21°01’55’’ |
|  | WZD2 | COLWZD2 | 20 | 109°05’59’’ | 21°02’01’’ |
| NZD | NZD1 | COLNZD1 | 20 | 110°37’06’’ | 20°54’19’’ |
|  | NZD2 | COLNZD2 | 20 | 110°35’34’’ | 20°54’22’’ |
|  | NZD3 | COLNZD2 | 20 | 110°35’37’’ | 20°54’38’’ |
|  | NZD4 | COLNZD3 | 18 | 110°33’46’’ | 20°54’06’’ |
| SCD | SCD1 | COLSCD1 | 20 | 112°46’55’’ | 21°43’50’’ |
|  | SCD2 | COLSCD2 | 20 | 112°47’02’’ | 21°43’41’’ |
|  | SCD3 | COLSCD2 | 20 | 112°47’01’’ | 21°43’43’’ |
| DAD | DAD1 | COLDAD1 | 20 | 113°43’12’’ | 22°01’22’’ |
|  | DAD2 | COLDAD2 | 20 | 113°43’07’’ | 22°01’19’’ |
|  | DAD3 | COLDAD3 | 20 | 113°43’06’’ | 22°01’16’’ |
|  | DAD4 | COLDAD4 | 20 | 113°43’12’’ | 22°01’29’’ |
| HBD | HBD1 | COLHBD1 | 20 | 113°10’25’’ | 21°52’25’’ |
|  | HBD2 | COLHBD2 | 20 | 113°10’16’’ | 21°51’45’’ |
|  | HBD3 | COLHBD3 | 20 | 113°10’30’’ | 21°51’49’’ |

Fig. S1a. The plot of the log-likelihood values for ten independent replications for k from 1 to 11 by using STRUCTURE.

Fig. S1b Mean logarithm of likelihood with variance for ten independent replications for k from 1 to 11 by using STRUCTURE. Error bars stands for standard deviations of ten runs.


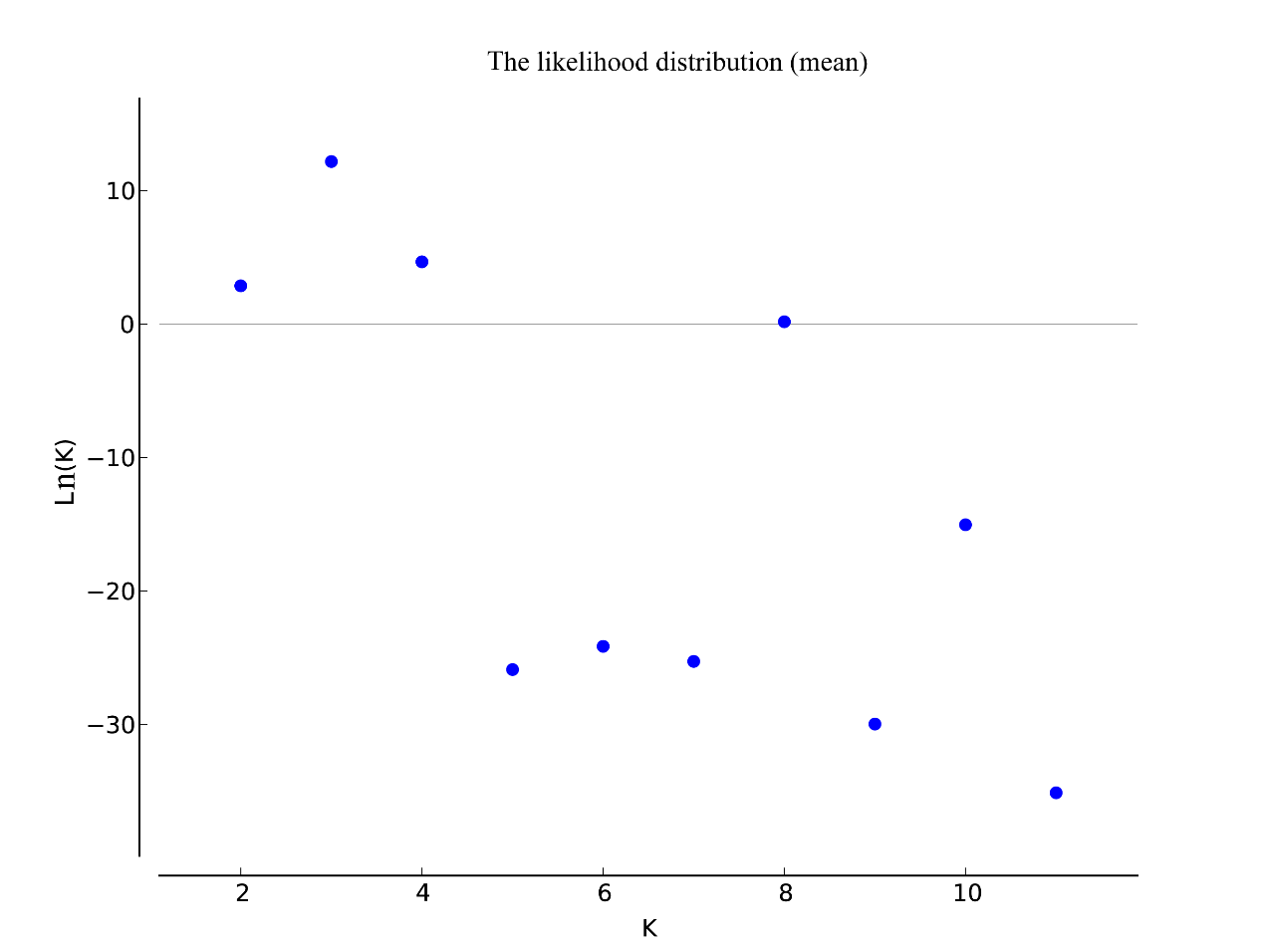


Fig. S1a


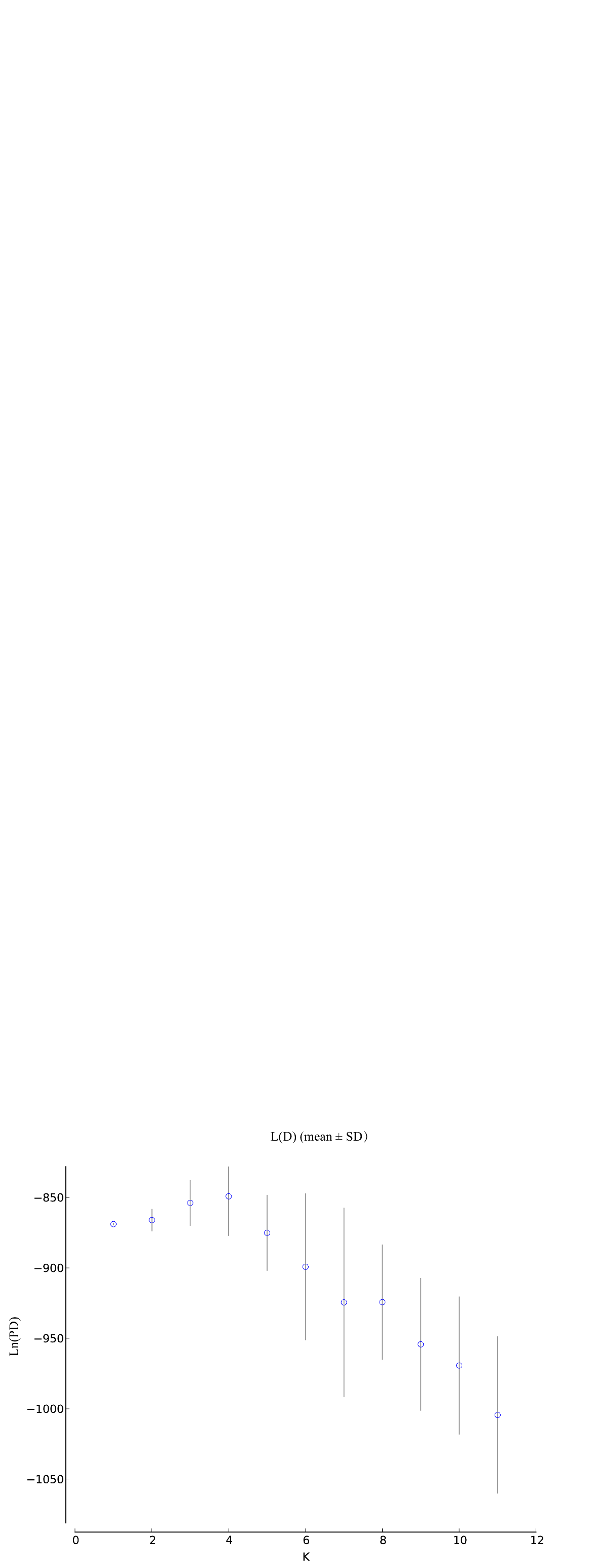


Fig. S1b
